# Modelling of pathogen-host systems using deeper ORF annotations and transcriptomics to inform proteomics analyses

**DOI:** 10.1016/j.csbj.2020.10.010

**Published:** 2020-10-14

**Authors:** Sebastien Leblanc, Marie A. Brunet

**Affiliations:** a Department of Biochemistry and Functional Genomics, Université de Sherbrooke, Sherbrooke, Québec, Canada; b PROTEO, Quebec Network for Research on Protein Function, Structure, and Engineering, Canada

**Keywords:** ORF, open reading frame, ncRNA, non-coding RNA, sORF, small open reading frame, ZIKV, Zika virus, HCMV, human cytomegalovirus, MS, mass spectrometry, AP-MS, affinity-purification mass spectrometry, PSM, peptide spectrum match, altProt, alternative protein, HCIP, highly confident interacting proteins, DEP, differentially expressed proteins, LFQ, label free quantification, FPKM, fragments per kilobase of exon model per million reads mapped, FDR, false discovery rate, Flavivirus, Zika, Alternative ORFs, Proteogenomics, Protein network, Proteome profiling

## Abstract

The Zika virus is a flavivirus that can cause fulminant outbreaks and lead to Guillain-Barré syndrome, microcephaly and fetal demise. Like other flaviviruses, the Zika virus is transmitted by mosquitoes and provokes neurological disorders. Despite its risk to public health, no antiviral nor vaccine are currently available. In the recent years, several studies have set to identify human host proteins interacting with Zika viral proteins to better understand its pathogenicity. Yet these studies used standard human protein sequence databases. Such databases rely on genome annotations, which enforce a minimal open reading frame (ORF) length criterion. An ever-increasing number of studies have demonstrated the shortcomings of such annotation, which overlooks thousands of functional ORFs. Here we show that the use of a customized database including currently non-annotated proteins led to the identification of 4 alternative proteins as interactors of the viral capsid and NS4A proteins. Furthermore, 12 alternative proteins were identified in the proteome profiling of Zika infected monocytes, one of which was significantly up-regulated. This study presents a computational framework for the re-analysis of proteomics datasets to better investigate the viral-host protein interplays upon infection with the Zika virus.

## Graphical abstract

**Figure:**
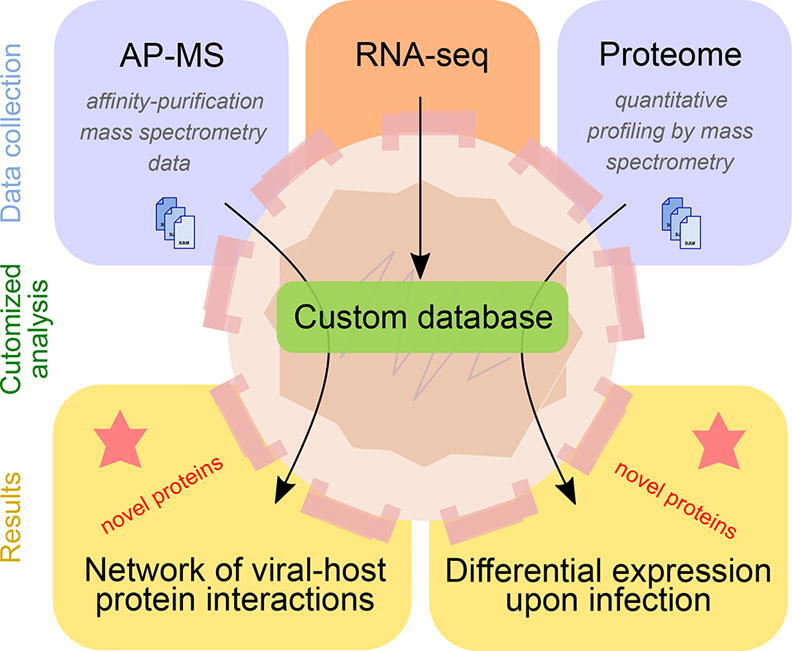

## 1 Introduction

Flaviviruses are the most common cause of arthropod-borne human diseases, such as dengue and yellow fevers, Japanese encephalitis, West Nile and Zika virus infections [1]. Among these, the Zika virus (ZIKV) has known a surge of interest from the scientific community following recent outbreaks [2], [3]. The first known outbreak took place on Yap Island in 2007, where 73% of the residents were infected and developed mild and short-lived symptoms [4]. The first association with more severe symptoms, such as Guillain-Barré syndrome, a neuro-inflammation of the peripheral nervous system, was discovered in the 2013–2014 outbreak in French Polynesia [5]. In late 2014, the virus spread through Brazil with a concomitant rise in Guillain-Barré syndrome and microcephaly in newborns [6]. Following these symptoms, the World Health Organization declared the Zika virus infection a Public Health Emergency of International Concern in 2016 [2].

ZIKV is an enveloped, positive-strand RNA virus. The primary source of infection is by mosquito bite, sexual and perinatal transmission [2]. ZIKV is blood-borne and can be detected 10 to 24 days post-infection in blood, and up to 60 days in semen [2]. However, the cellular and molecular intricacies of ZIKV pathogenesis, especially how the virus rewires the cellular pathways in favor of its replication, are not completely understood. Recently, several groups have used transcriptomics and proteomics methods to identify up- or down-regulated proteins during Zika virus infection, and to decipher its interaction with host proteins [3], [7], [8], [9], [10], [11], [12]. These studies are pivotal as they identify regulated pathways and protein–protein interactions between the host cell and the virus. However, and as reviewed in [3], such datasets are complex and findings are for the most part not reproduced across studies. First, comparisons across networks of viral-host protein interactions revealed a poor overlap [3]. These discrepancies certainly reflect experimental specificities such as the viral strain, the cellular background, the gene-delivery method and the experimental design. However, an often-overlooked source of variabilities is the analytical method used, both for the analysis and for the comparison. Furthermore, comparisons of RNA-seq and proteomics-based data in Zika-infected cells also revealed large differences. These indicate that the protein abundances cannot be confidently inferred from mRNA expression levels [13]. Although this is also true for physiological states, it is particularly relevant during infections as viruses modulate gene expression in part by altering mRNA processing, transport and translation rate [14], [15], [16].

Interestingly, viral infection changes the translational landscape of the host cell [15], [16]. These translational changes can lead to the production of novel unannotated proteins. For example, in cells infected with the human cytomegalovirus (HCMV) several small open reading frames (sORFs) from the β2.7 allegedly non-coding RNA (ncRNA) were translated [17]. Sera samples from HCMV-positive blood donors revealed a strong response to these sORFs-encoded peptides, suggesting expression of these proteins and presentation on MHC molecules as functional antigens [18]. Additionally, a genetic screen study identified a novel protein, CYREN, encoded in a previously annotated ncRNA, among peptides important for resistance to retroviral infection [19]. More recently, a large-scale study identified 19 novel proteins, encoded by non-annotated ORFs in the human genome, as differentially regulated upon infection with flu lysates [20]. These findings highlight how current genome annotations shape the investigations of viral-host protein interplays.

The shortcomings of current annotations have been increasingly demonstrated throughout the last decade [21], [22], [23]. In order to minimize the identification of random non-functional ORFs, genome annotations enforce two arbitrary criteria : a minimal length of 100 codons and a single ORF per transcript, except for previously characterized examples [21]. However, these criteria are not supported by experimental evidence and led to an oversight of small and alternative ORFs [24], [25], [26]. Alternative ORFs are found in regions currently annotated as non-coding (ncRNAs, 5′ and 3′ UTRs of mRNAs) or overlapping an annotated ORF in a different reading frame [21], [27]. To foster a more systematic exploration of alternative ORFs, several repositories have been published [28], [29], [30]. Such resources are pivotal for a deeper exploration of cellular events [21], [26], [31]. For example, in mass spectrometry (MS)-based proteomics, mass spectra must be matched to theoretical spectra generated from a database of possible proteins during the analysis. Thus, if a protein is absent from the database, it cannot be identified [21]. Differences in the database used for the analysis of MS data to elucidate the viral-host protein interactions can also be a source of discrepancies across studies. More importantly, it could prevent the identification of key interactors that are currently not annotated [17], [19], [20], [32].

In this study, we propose a computational framework to investigate the importance of non-annotated proteins in the Zika infection through the re-analysis of published MS and RNA-seq datasets. We first address the comparison between such complex datasets and validate our MS analysis pipeline to identify highly confident interacting proteins (HCIPs). We then take advantage of published RNA-seq data to build a custom database using annotated proteins (UniProtKB, Ensembl and NCBI RefSeq) [33], [34], [35] and alternative proteins (OpenProt) [30]. Using this custom database, we queried an affinity-purification MS (AP-MS) dataset and a proteome profiling dataset for novel proteins important in the Zika virus pathogenicity. This computational framework demonstrates the importance of transcriptomic-informed proteomic analyses to identify changes in transcriptomic, translational and proteomic landscapes upon ZIKV infection.

## 2 Materials & Methods

### 2.1 Mass spectrometry-based proteomics

#### 2.1.1 Datasets and databases

Affinity-purification mass spectrometry (AP-MS) data originated from a study by Shah and colleagues [8], and was retrieved from the Chorus repository (accession Project ID 1438). This dataset was generated using HEK293 cells infected with 2 strains of Zika virus (ZIKV French Polynesia 2013H/FP/2013 and ZIKV Uganda 1947 MR766). The experimental procedures have been described in the original study [8]. Briefly, Zika virus ORFs were tagged with a C-terminal 2xStrep II affinity tag and inserted into a pCDNA4_TO plasmid for expression in HEK293 cells. The authors used Strep-tactin beads to purify viral proteins and their interactors 40 h post-infection. The samples were digested with trypsin overnight before analysis on a Q-Exactive Plus Orbitrap (ThermoFisher) mass spectrometer.

Whole proteome label-free quantification data originated from a study by Ayala-Nunez and colleagues [9], and was retrieved from the PRIDE repository (accession PXD014002). This dataset was generated using human monocytes purified from 2 healthy blood donors. The experimental procedures have been described in the original study [9]. Briefly, cells were either infected with the Zika virus or not infected and quantitative proteomic profiling was performed 48 h after infection using a Q-Exactive Plus Orbitrap (ThermoFisher) mass spectrometer.

For annotated proteins, sequences were retrieved from the UniProtKB resource (*Homo sapiens* SwissProt, 2020–03). This fasta file contained 20,352 proteins. Protein sequences from the Zika virus were appended as described in [8]. The custom database was built using RNA-seq data to filter the whole OpenProt database (version 1.5: a non-redundant list of proteins from the full UniProtKB (03–2019), Ensembl (Jan 2019) and NCBI RefSeq (Jan 2019) databases as well as novel predicted proteins). Thus, our custom database contained 98,508 protein sequences. These included 13,048 proteins from the full UniProtKB database, 83 proteins from Ensembl 95 and 311 proteins from NCBI RefSeq not contained in the UniProtKB database, 4,469 novel isoforms from the OpenProt database (version 1.5, 2020–06), 80,573 alternative proteins from the OpenProt database, and 19 protein sequences derived from the RNA-seq data (see Section 2.3.2).

#### 2.1.2 Mass spectrometry analysis pipeline

Raw AP-MS files were first converted to mgf files using the ThermoRawFileParser (version 1.2.0) [36]. The files were analysed using PeptideShaker software (version 1.16.42) [37] configured to use three search engines (X!Tandem, MS-GF+ and Comet) via SearchGUI (version 3.3.17) [38]. The decoy database was generated using reversed sequences. SearchGUI general parameters were set as follows: the fragment mass tolerance was set to 20 ppm and the precursor ion tolerance to 4.5 ppm; the enzyme was set to trypsin with a maximum of 2 missed cleavages; oxidation of methionine and acetylation of protein N-terminus were set as variable modifications, and carbamidomethylation of cysteine was set as fixed modification; a maximum of 5 modifications were allowed per peptide, a maximum charge of 7 + and minimal length of 7 amino acids. False discovery rates (FDR) were set to 1% at the peptide and protein level. Additionally, a novel protein was deemed confidently identified only if supported by at least one unique peptide. Thus, similarly to the OpenProt pipeline, the following peptide assignation rules were enforced: if a peptide was shared between 2 known proteins the spectrum was assigned to both and a protein group was created; if a peptide was shared between 2 novel proteins the spectrum was assigned to both, and a protein group was created; if the peptide is shared between known and novel proteins, the spectrum was only be assigned to the previously known proteins. Although biased against novel proteins, this approach ensured a certain robustness in their identification.

For quantitative proteome profiling, raw files were analysed with MaxQuant (version 1.6.0.16) [39]. The decoy database was generated using reversed sequences. The search parameters were set as follows: the fragment mass tolerance was set to 20 ppm for the first search and 5 ppm for the main search; MS/MS tolerance was set to 40 ppm; the enzyme was set to trypsin with a maximum of 1 missed cleavages; oxidation of methionine was set as variable modification, and carbamidomethylation of cysteine as fixed modification; label-free quantification (LFQ) was implemented using a minimal ratio count of 1 [40]; and the match between runs was enabled with a 2-min time window after retention-time alignment. The protein FDR was set to 1%, with the same peptide assignation rules as the AP-MS data analysis.

### 2.2 Protein interaction and quantification analyses

#### 2.2.1 Protein interaction scoring with MiST and CompPASS

Protein-protein interaction candidates from the AP-MS analysis were scored using both MiST [41] and CompPASS [42] scoring algorithms. First, to control for data quality and reproducibility across replicates, peptide and protein counts per sample, as well as bait spectral counts were used to screen unreliable samples. At least three replicates for each bait were kept.

For MiST scoring, peptide spectrum match (PSM) counts were used as quantifying feature. The recommended weights relating to reproducibility (R), bait specificity (S) and abundance (A) of the interaction were used: R = 0.309, S = 0.686 and A = 0.006 [41], [43]. Only proteins with a MiST score above 0.75 were considered when using MiST alone to score highly confident interacting proteins (HCIPs). For CompPASS scoring, PSM counts were also used as quantifying feature. When using CompPASS alone to score HCIPs, proteins with a weighted D-score in the top 5% were considered. To evaluate the performance of each HCIP scoring protocol, the proteins identified in the original study were used as target true positives [8]. Thus, the precision was calculated as: $\frac{\mathrm{Truepositive}}{\mathrm{Truepositive}+Falsepositive}$ . The recall was calculated as: $\frac{\mathrm{Truepositive}}{\mathrm{Truepositive}+Falsenegative}$ . The F1 score was calculated as: $2\times \frac{\mathrm{Precision}\times Recall}{\mathrm{Precision}+Recall}$ . The precision, recall and F1 score calculated for each approach is shown in Supplementary Fig. 1.

#### 2.2.2 Naive Bayes classification

To further optimize prey hits filtration (optimize the F1 score) and to capitalize on the optimization work from the original study [8], a Naive Bayes model was trained. Such classifiers have previously been published to identify HCIP in large datasets [44]. The model was trained to distinguish HCIP from background proteins using interactions from the original study and another AP-MS experiment with ZIKV viral proteins in HEK293 cells [11] as positive labels. MiST score and the CompPASS Z-score were used as features. We used the CompPASS Z-score as it complemented the MiST score whereas the weighted D-score correlated with it. To avoid over-fitting, the set of candidate interactions was randomly divided into 10 sets and each was scored with a classifier trained on data from the other nine sets in a 10-fold cross-validation manner. Thus, 10 models were trained, each blinded to the set of interactions it was aimed to evaluate. The model assigned a score reflecting the likelihood of a specific interaction for each bait-prey pair. The precision, recall and F1 score were calculated as described above using interactions from the original study as labels for positive HCIPs. The threshold for the Naïve Bayes was selected by observing the behavior of the three metrics across a range of thresholds. The threshold that optimized F1 score while prioritizing recall over precision (Supplementary Fig. 1). The inherent lack of true negative examples in protein interaction datasets requires prioritizing recall over precision when selecting the classifier threshold. It is generally expected that the rate of false positives be over estimated due to this bias. Thus, a threshold of 0.45 was chosen for the analysis with the UniProt database, and 0.42 for the analysis with the custom database (Supplementary Fig. 2).

#### 2.2.3 Protein quantification and differential expression analysis

LFQ intensities were reported by MaxQuant and used for quantification and differential expression analysis. To be included in the analysis, proteins needed be identified in all replicates (4 replicates) in at least one condition (4 conditions: 2 non-infected and 2 Zika-infected). For known proteins, a requirement for a minimum of two unique peptides was also enforced. For novel proteins, considering their smaller size (Fig. 3) and the bias inherent to the peptide assignation rules (see Section 2.1.2), a minimum of one unique peptide was used. Missing values were imputed using the ProStaR software (version 1.18.6) [45] as done in the original study. Briefly, the imputation was done differentially based on the nature of the missing values. We used a structured least square adaptative regression (SLSA mode) for partially observed values (POV), while a deterministic value (DetQuantile method – 1% quantile, multiplying factor of 1) was applied for values missing in the entire condition (MEC). The DetQuantile method was chosen for MEC as these values corresponded to proteins below the limit of detection in one condition, thus they could not be imputed based on values observed in other conditions [45]. No data distortion could be observed after imputation of missing values (Supplementary Fig. 6). Differential expression analysis was performed using a Limma-moderated *t*-test with a Benjamini-Hochberg correction for multiple comparisons. The FDR was set to 1% for downstream analyses.

### 2.3 Transcriptomics analysis

#### 2.3.1 RNA-seq dataset

The RNA-seq data originates from a study by Tang and colleagues [10], and was retrieved from the NCBI Gene Expression Omnibus (GEO) repository (accession GSE78711). This dataset was generated using human cortical neural progenitor cells (hNPCs) that were either non-infected or infected with Zika virus (ZIKV Uganda 1947 MR766 strain) at low multiplicity of infection (MOI) (<0.1). The experimental procedures have been detailed in the original publication [10]. Cells were collected 56 h after infection. The dataset includes 2 replicates for each condition, and each was paired-end sequenced.

#### 2.3.2 RNA-seq data analysis

We used the FastQC toolkit (version 0.11.8) with default parameters to filter low quality reads. The reads were trimmed using TrimGalore (version 0.6.4) set for paired reads with the default parameters except for the maximal number of N set to 5, and end clips set to 3. The reads were subsequently mapped to the human genome (GRCh38, Gencode v32, primary assembly) using the STAR software (version 2.7.3a) [46] with the default parameters except for the maximal number of mismatch set to 5, the maximal number of multi-mapping locations set to 10 and SAM primary flag set to all best scores. The outputs were ordered by genomic coordinates. Cufflinks (version 2.2.1) [47] was used for transcript assembly and evaluation of the transcript expression level (FPKM - fragments per kilobase of exon model per million reads mapped). For inclusion in the custom database, transcripts must be identified in both replicates of the Zika-infected condition and have a FPKM above 2.8 (see Fig. 3).

### 2.4 Novel protein predictions

#### 2.4.1 OpenProt resource mining

Novel proteins were retrieved from the OpenProt resource [30]. We used version 1.5 of the OpenProt database for *Homo sapiens* (06–2020). These predictions were based on the GRCh38.p12 genome assembly and contain all ORFs, currently not present in annotations, longer than 30 codons and starting with an ATG. Subsequently, the corresponding proteins were classified as novel isoforms or alternative proteins based on their homology (or lack thereof) with the canonical protein from the same gene. The Fasta files containing alternative proteins and novel isoforms were downloaded using the downloads interface (www.openprot.org). For downstream analyses, the search interface was used to gather previous experimental evidence, conservation and prediction of functional domains for novel proteins.

#### 2.4.2 ORF prediction on novel transcripts

For novel transcripts predicted by Cufflinks and with enough supporting evidence (detected in both replicates with a FPKM above 1.499), ORFs were predicted by *in silico* translation using the same criteria as those implemented by OpenProt: a minimal length of 30 codons and an ATG start. Each transcript was assigned an accession number (tx0000) preceded by the string “CUFF_zika” and each ORF from a given transcript was assigned a unique number. Each ORF was then searched within the OpenProt database. If the exact same ORF exists in the OpenProt database from another transcript from the same gene, the encoded protein was given the accession listed by OpenProt. When the ORF was not present in the OpenProt database (novel ORF), the resulting protein accession corresponded to the transcript accession concatenated to the ORF number to provide a unique identifier for each predicted ORF.

### 2.5 Network analyses

#### 2.5.1 Network similarity measures

Similarity of networks can be measured at two levels: similarity of identified interacting proteins, and similarity of protein complexes identified. For the overlap of identified interacting proteins, the list of HCIPs for each network is compared using the Jaccard similarity index [48] which considers the ratio of size of the intersection of both sets to the size of the union. The significance of this overlap is then evaluated with a Fisher’s exact test. A p-value below 0.05 was considered significant. To evaluate the similarities between protein–protein interaction networks from different analyses, we measured network characteristics: degree, local clustering coefficient, and shortest path length distributions. In a protein–protein interaction network, the nodes correspond to proteins and edges represent interactions. The degree of a node is defined as its number of connections. The local clustering coefficient relates to the interconnectivity of the neighborhood of a node with higher values indicating denser connectivity. The shortest path length is defined as the minimal number of edges required to connect two nodes on the network. These metrics were calculated for each protein (degree and clustering coefficient) or pair of proteins (shortest path).

#### 2.5.2 Biological processes enrichment

Enrichment of biological processes was measured using the GOATOOLS Python package (version 1.0.2) [49], corresponding to the GO-term annotation version 1.2. The GOATOOLS package was run with the following parameters: alpha = 0.05, and count propagation to the parental terms set to true. The enrichment was calculated against the human proteome for each bait with a Benjamini-Hochberg correction to adjust for multiple comparisons. The FDR was set to 5%.

#### 2.5.3 Edge mapping from STRING database

Known interactions between proteins within the network were retrieved from the STRING database (version 11.0). Only interactions with a combined score of 0.75 (considered highly confident) were retrieved.

#### 2.5.4 CORUM complexes overlap

The similarity of networks from different analyses was also considered via the retrieval of CORUM complexes. Briefly, three sets of complexes with at least one protein identified in the original study [8], our analysis, or both, were retrieved from the CORUM database (version 3.0). The number of subunits identified was then calculated for each identified complex.

#### 2.5.5 Code and data availability

The AP-MS data was retrieved from the Chorus repository (www.chorusproject.org, Project ID 1438). The quantitative proteome profiling was retrieved from the PRIDE repository (https://www.ebi.ac.uk/pride/archive/, PXD014002). The RNA-seq data was retrieved from the GEO database (https://www.ncbi.nlm.nih.gov/geo/, GSE78711). The human genome assembly was retrieved from the Gencode server (https://www.gencodegenes.org/, v32 primary assembly). Alternative protein and novel isoform sequences were retrieved from the OpenProt resource (https://openprot.org/, version 1.5 06–2020). All scripts were written with Python 3.7 and Networkx 2.4, and are available in GitHub (https://github.com/MAB-Lab/Zika_Project).

## 3 Results

### 3.1 Assessing reproducibility from AP-MS data re-analysis

**Fig. 1:**
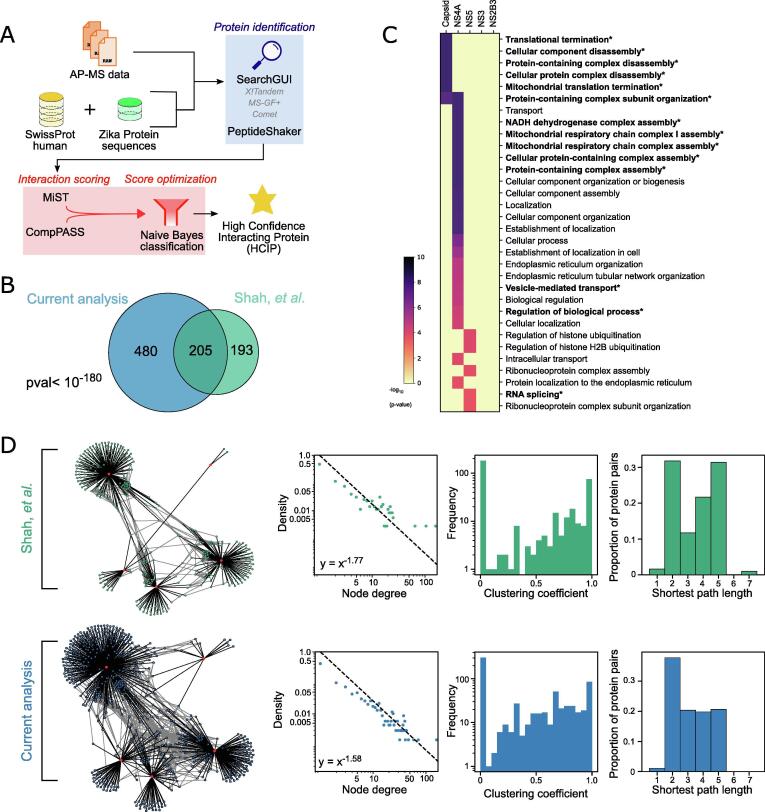
Evaluating reproducibility across AP-MS data analysis A. Graphical representation of the pipeline used for affinity-purification mass spectrometry (AP-MS) data analysis. AP-MS data is interrogated using a combined database of human protein sequences (yellow) and Zika viral protein sequences (green). The pipeline contains a step of protein identification (blue) using SearchGUI and PeptideShaker, followed by interaction scoring (red) using MiST, CompPASS and a Naïve Bayes classification algorithm, to produce a list of highly confident interacting proteins (HCIPs). B. Overlap of proteins identified in this study (current analysis) with that of the original study from Shah and colleagues [8]. Significance of the overlap was evaluated using a Fisher’s exact test. C. Gene ontology (GO) enrichment of interacting proteins (preys) for each viral proteins (baits – top). The heatmap displays all significant enrichments (FDR < 1%, Benjamini-Hochberg correction). The GO terms highlighted in bold and a star correspond to GO terms also identified in the original study. D. Network characteristics between the current analysis and that of the original study. Networks are represented with bait proteins in red (Zika proteins) and interacting proteins in green for the original study and in blue for the current analysis. The plots from left to right correspond to the node degree distribution, the distribution of local clustering coefficients and the distribution of shortest pair length. (For interpretation of the references to color in this figure legend, the reader is referred to the web version of this article.)

The most-used technique to build a network of protein interactions is the AP-MS technique [50], [51]. “Bait” proteins are expressed with a tag which allows purification and subsequent identification of interacting proteins (“preys”) by MS [3]. Here, we retrieved a publicly available AP-MS dataset of Zika viral proteins in human cells [8]. As in the original study, we used the SwissProt database concatenated with sequences of the Zika proteins as a reference protein database. However, our analytical pipeline used a different strategy for the protein identification and interaction scoring strategies (Fig. 1A). For the protein identification step, we used the SearchGUI and PetideShaker softwares to take advantage of multiple search engines [52]. We identified 2,490 unique potential interactors, from which 1,762 (70.8%) were also identified in the original study (Suppl. Fig. 1). Out of the 277 proteins found in the original study but not in our analysis, 4 were not present in the SwissProt database we used (03–2020) and 196 were found within protein groups but were not selected as they were not supported by unique peptides in our hands (Suppl. Fig. 1). These numbers demonstrate the importance of databases and protein group handling in AP-MS analyses.

To identify highly confident interacting proteins (HCIPs) from background interactions, we tested 4 filtering protocols: the MiST algorithm alone [51], the CompPASS algorithm alone [42], the intersection of MiST and CompPASS calls, and the use of a Naive Bayes classifier building on MiST and CompPASS scores. Using MiST alone did not produce enough filtering of our dataset (1,501 HCIPs called, corresponding to the top 12.1% of interactions kept), where CompPASS alone sufficiently filtered but yielded a poor recall (28%) and F1 score (25%) (Suppl. Fig. 1). The intersection of both scoring methods improved the F1 score (31%) but still yielded a poor recall with a stringent filter (274 HCIPs called, corresponding to the top 2.2% of interactions kept). The Naïve Bayes built on the strength of both MiST and CompPASS reaching the highest F1 score (40%) with a reasonable recall (57%) and calling a total of 665 HCIPs for a threshold of 0.45 (Suppl. Fig. 1).

Out of these 665 HCIPs, 199 were also identified in the original study [8] (Suppl. Table 1). Thus, our pipeline was able to identify 56.7% of the protein–protein interactions identified in the original study (Fig. 1B). Although this overlap might not seem large, it is highly significant (Fisher’s exact test, p-value < 10^-180^). As mentioned in the introduction, discrepancies between AP-MS datasets have been reported previously [3]. We show here that such discrepancies can originate from the computational analysis of the raw data. That is in part because AP-MS techniques pull on protein complexes, which often share peptides across subunits or elements of a complex. The method by which the search algorithm and downstream analyses handle the protein groups skews the identification towards particular subunits or members of a protein complex. Hence, to verify the validity of our results we calculated the enrichment of biological processes from our 665 HCIPs (Fig. 1C). All GO terms identified have been reported previously in association with viral infection, and 14 terms were also reported in the original study. Interestingly, we noticed that both analyses agreed on the most significant GO terms (11 out of 16 GO terms with a p-value below 0.00005, Suppl. Table 2). We hypothesized that our analysis identified the same biological complexes than those in the original study, but different proteins within these complexes. To test this hypothesis, we retrieved the network published by Shah and colleagues and compared it with the network generated from our analysis (Fig. 1D). Baits are positioned identically to ease the visual comparison. We observe that the network from the current analysis is more interconnected than that of the original study. Relatively densely interconnected networks are expected when looking at protein–protein interactions [44], [53], [54]. As expected of naturally occurring networks node degree distributions fitted a power law. The distribution from the original study however seemed to deviate more, especially towards higher degrees (Fig. 1D). Local clustering coefficient distribution from the current analysis indicated a more coherent subnetwork region of the human whole interactome likely involved in ZIKV activity. Perhaps because of excessive stringency, the network from the original publication may have overlooked peripherally located yet important proteins in ZIKV’s effect on system shift towards a regime geared for its replication. Indeed, the original study reports only the most densely connected regions of the network and likely missed protein interactors more relevant to rewire connections between complexes necessary for viral hijacking of cellular processes [54]. The sparsity of the network from the original study is also visible from the bimodal distribution of the shortest path between all protein pairs. While the network from the current analysis still shares a significant homology (i.e. relative number of preys for each baits) with that of the original study (Fig. 1D), the more continuous distribution in shortest path lengths shows structure closer to that expected of typically interconnected protein networks and may provide a more holistic model of ZIKV infection. Furthermore, protein complexes from the CORUM database with subunits found in the original study and the current analysis were recovered more reliably in the current study. 132 complexes were found in both analyses (72.7%), with the current analysis identifying more subunits within these complexes (Suppl. Fig. 1).

### 3.2 Zika viral protein interactions reveal viral host-mRNA translational control

**Fig. 2:**
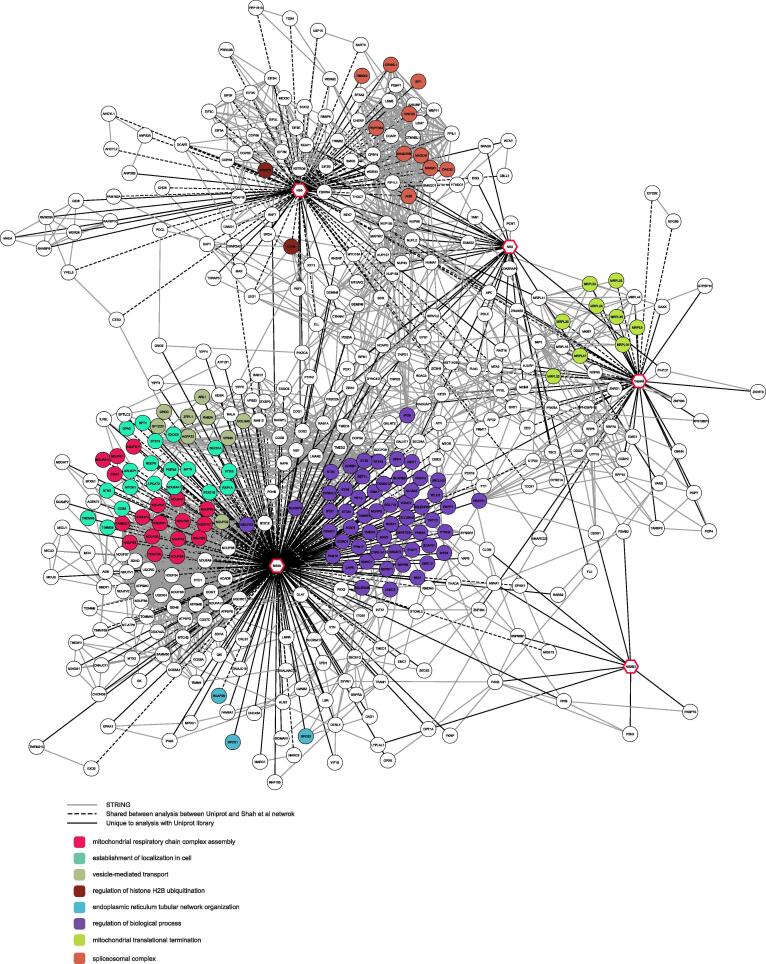
Network of viral-host protein–protein interactions Network of protein interactions for proteins of the Zika virus (indicated as octogonal nodes with thick red borders) with proteins with a degree higher than 1. The nodes (circle with black borders) correspond to human proteins identified by AP-MS data analysis. Nodes are coloured based on their associated gene ontology as indicated on the bottom left corner. Edges represent confident interactions between two proteins as follows: solid black lines are interactions only identified in the current analysis; dashed black lines are interactions shared with the original study (Shah, *et al*.); and full grey lines are host–host protein interactions retrieved from the STRING database. (For interpretation of the references to color in this figure legend, the reader is referred to the web version of this article.)

We built a network incorporating our HCIPs and highlighted the proteins also identified in the original study (for brevity Fig. 2 represents only proteins with a degree above 1, see Suppl. Fig. 3 for whole network). For the capsid, NS5 and NS4A viral proteins, both networks agreed on protein complexes. For some the same proteins have been identified (dashed black edges, Fig. 2, Suppl. Fig. 3), as it is for the translational termination complex (MRPL9 (Q9BYD2), MRPL20 (Q9BYC9), MRPL23 (Q16540) and MRPL47 (Q9HD33)) or the spliceosome (AQR (O60306), CWC25 (Q9NXE8), PRPF38A (Q8NAV1) and SRRM1 (Q8IYB3)). For others different members of a same complex were identified (solid lines), as it was for the mitochondrial translational termination complex (in green on Fig. 2) or the mitochondrial respiratory chain complex assembly (in pink on Fig. 2). Both networks diverge mostly on protein identifications for the NS3 and NS2B3 (Fig. 2). In both analyses, no confident GO term enrichment was found for NS2B3. In the original study, the NS3 interactome was found enriched for the GO term “Spindle formation” driven by a single protein (HSPA2 (P54652)). That protein is also identified in our analysis, however we filtered out enrichment driven by a single protein. In total, we identified 14 proteins interacting with NS2B3 and 67 proteins interacting with NS3 (Suppl. Table 1).

Interestingly, in agreement with the published literature on flaviviruses, 35% of GO terms identified in our analysis relate to mRNA processing, splicing, transport and translational control (Fig. 2). These associations were mostly linked to the NS5 and capsid viral proteins, as previously reported [7], [8], [11]. With previous studies having highlighted pervasive translation and the biological importance of some alternative proteins upon viral infection [17], [18], [20], we hypothesized that Zika viral proteins may also interact with proteins currently missing from protein databases.

### 3.3 Building a custom database for deeper proteome exploration

**Fig. 3:**
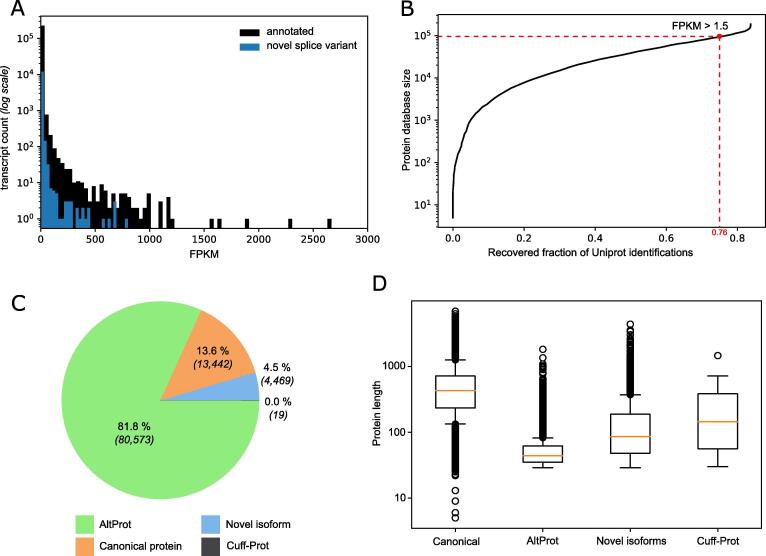
Construction and specifics of the custom database A. Distribution of transcripts identified in the RNA-seq analysis by their estimated level of expression (FPKM). Annotated transcripts are indicated in black, while novel transcripts are indicated in blue. B. Relation curve between the size of the custom database and the fraction of UniProt identifications included in the custom database, based on the FPKM threshold from the RNA-seq analysis. The chosen FPKM threshold (1.5) is indicated by a red dot. C. Composition of the custom database. Proteins are classified as canonical proteins if annotated in UniProt, NCBI RefSeq and/or Ensembl (orange). Proteins are otherwise called novel isoforms (blue), alternative protein (altProt - green) of CUFF-transcript derived protein (Cuff-Prot - grey). D. Distribution of the protein length for each protein category defined in panel C. Boxes represent the inter quartile range (IQR) notched at the median and the whiskers are set at 0.5*IQR over and under the 25th and 75th percentiles. (For interpretation of the references to color in this figure legend, the reader is referred to the web version of this article.)

One caveat of inclusion of alternative proteins in databases for proteomics experiments is the consequent increase in the size of the resulting database. Large databases lead to a decrease in the specificity and sensitivity of the analysis, resulting in a low number of confidently identified proteins and a higher rate of false positives [55], [56]. Thus, it is recommended to use adapted pipelines, such as a stringent 0.001% false discovery rate (FDR), or to limit the size of the database to a maximum of 100,000 entries [30], [32], [57]. Here, we retrieved RNA-seq data to identify transcripts confidently expressed in Zika-infected cells and build a custom database tailored to a Zika infection context. Since the Zika virus leads to alternative splicing of transcripts [58], as supported by the AP-MS data analysis (in orange on Fig. 2), we used a pipeline to identify both canonical transcripts and novel splice variants (Fig. 3A). Most transcripts displayed an expression level below 200 FPKM, with a few highly expressed transcripts (Suppl. Table 3). The custom database is designed to include any protein (canonical, alternative protein, novel isoform, and Cuff-Prot – defined in Table 1) from transcripts detected by RNA-seq above a certain expression (FPKM) threshold. To select the optimal threshold, we evaluated the size of the resulting custom database and the proportion of UniProt proteins identified in our previous analysis (Suppl. Table 1) included in the database (Fig. 3B). We identified 1.5 as the ideal FPKM threshold at which 76% of proteins identified in our first analysis were included in the resulting database, while maintaining an overall database size below 100,000 proteins (Suppl. Data 1).

**Table 1:** Definition of categories of proteins in the custom database.

Name	Full name	Description	Source
Canonical	Canonical protein	Protein annotated in current annotations	UniProtKB, Ensembl and/or NCBI RefSeq
AltProt	Alternative protein	Protein currently not annotated with no significant homology with the canonical protein of the same gene	OpenProt
Novel isoform	Novel protein isoform	Protein currently not annotated with a significant homology with the canonical protein of the same gene	OpenProt
Cuff-Prot	Cuff-transcript derived protein	Predicted protein from an ORF within a novel splice variant transcript detected by RNA-seq	*In silico* translation

The resulting custom database contained 98,508 proteins (Fig. 3C). These included 13,442 canonical proteins, detailed as 13,048 proteins from the full UniProtKB resource [33], 83 proteins from the Ensembl annotation [34] and 311 from the NCBI RefSeq annotation [35] not present in the UniProtKB database. The database also contained 80,573 alternative proteins and 4,469 novel isoforms of canonical proteins from the OpenProt resource [30]. Finally, 19 protein sequences were derived from novel splice transcripts identified in the RNA-seq analysis. Since genome annotations enforce a minimal length criteria of 100 codons, except for previously characterized examples, this introduced a protein length bias across the different protein categories (Fig. 3D), as previously observed [26]. AltProts displayed the lowest median length (44 amino acids), while novel isoforms and Cuff-Prots displayed a slightly increased median length (86 and 145 amino acids respectively) although still much shorter than that of the canonical proteins (429 amino acids).

### 3.4 AP-MS data analysis with a custom database retrieves confident canonical interactions

**Fig. 4:**
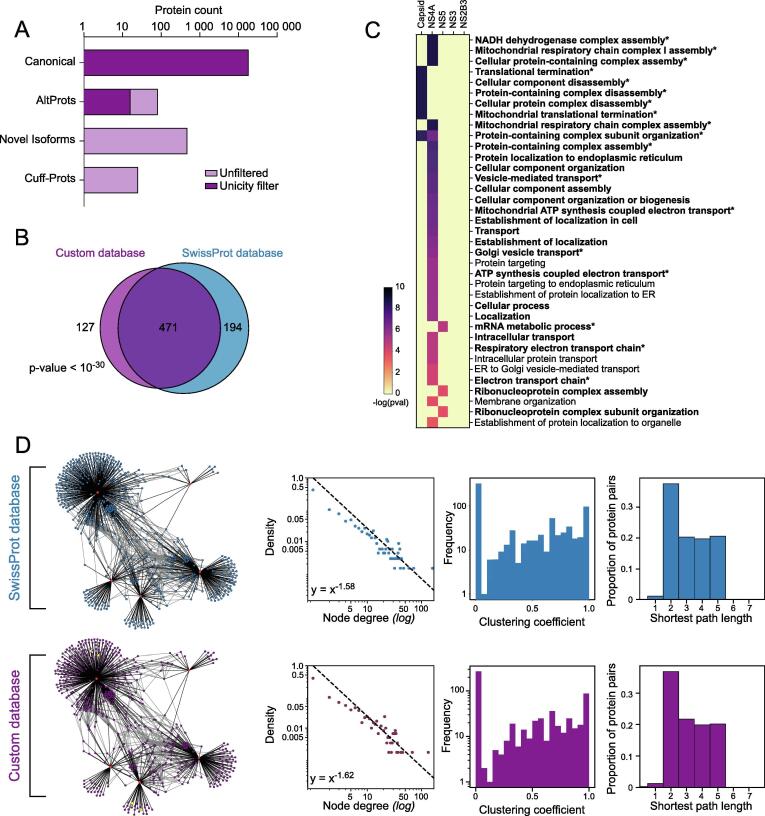
AP-MS data analysis features with the custom database A. Count of proteins identified in the protein identification step of the analysis for each protein category (canonical, altProt, novel isoforms and Cuff-Prots). The light purple corresponds to the counts prior to the peptide unicity additional filter. The dark purple corresponds to the counts after filtering for peptide unicity across the whole proteome. B. Overlap of proteins identified with the custom database or the SwissProt database (see Fig. 1). Significance of the overlap was evaluated using a Fisher’s exact test. C. Gene ontology (GO) enrichment of interacting proteins (preys) for each viral proteins (baits – top) identified with the custom database. The heatmap displays all significant enrichments (FDR < 1%, Benjamini-Hochberg correction). The GO terms highlighted in bold and a star correspond to GO terms also identified with the SwissProt database. D. Network characteristics between the current analysis and that of the original study. Networks are represented with bait proteins in red (Zika proteins) and interacting proteins in blue for the analysis with SwissProt and in purple for the current analysis. Alternative proteins are indicated in yellow. The plots from left to right correspond to the node degree distribution, the distribution of local clustering coefficients and the distribution of shortest pair length. (For interpretation of the references to color in this figure legend, the reader is referred to the web version of this article.)

We re-analysed the AP-MS dataset from Shah and colleagues [8] with our custom database concatenated with the Zika viral protein sequences. Prior to HCIP filtering, we identified across all replicates for all baits 18,258 interactions with canonical proteins, 81 with altProts, 472 with novel isoforms and 25 Cuff-Prots (Fig. 4A). However, as usual in standard MS-based proteomics, the concept of peptide unicity is inherent to the database used. Here, we want to report novel proteins only if the peptide cannot be explained by a canonical protein, including those not present in the custom database (i.e. due to low transcript abundance). Thus, we enforced a second peptide unicity check against the whole OpenProt database (version 1.5). This left us with 18,258 interactions with a canonical protein and 16 with an altProt (Fig. 4A). It is to note that such peptide assignation rules create a bias against the detection of novel proteins, although the detections are more confident. After parsing for detection in at least two replicates, we obtained a list of 1,954 identified proteins, corresponding to 9,770 potential interactions, which included 7 altProts.

598 HCIPs were ultimately identified using the custom database (Fig. 4B, Suppl. Table 4). Out of these 598 HCIPs, 471 were also identified in the analysis with the SwissProt database. Thus, the pipeline was able to identify 70.8% of the proteins identified with the SwissProt database. This overlap is both highly significant (Fisher’s exact test, p-value < 10^-30^) and underestimated: since only 76% of the SwissProt protein entries identified in the first analysis were included in the custom database based on the RNA-seq analysis, the overlap is of 93.1%. Furthermore, the GO enrichment analysis revealed a strong overlap with the analysis using the SwissProt database (30 of the 37 terms – Fig. 4C, Suppl. Table 5). The new terms found with the custom database are particularly relevant to viral infection. Notably, the custom database identified proteins involved in SNAREs-mediated membrane fusion events (USE1 (Q9NZ43), BET1 (O15155), COG3 (Q96JB2) and GOSR2 (O14653)) as interactors of the viral NS4A protein. These proteins were identified in the analysis with the SwissProt database but scored just under the threshold of the Naïve Bayes (Suppl. Table 1). This relates to studies in other flaviviruses that found NS4A to be implicated in the formation of replication factories, organelle-like membranous structures resulting from drastic re-organization of ER membranes [14], [59]. Furthermore, proteins associated with the nuclear chromatin were enriched amongst Capsid interactors (YY1 (P25490), HIST2H2BE (Q16778), BAZ2A (Q9UIF9), and SMARCD2 (Q92925)). Interestingly, all of these have been found interacting with the sirtuin protein (SIRT1), and sirtuin inhibitors were reported to block Zika virus infection downstream of viral entry [60]. These proteins were not identified in the analysis with the SwissProt database (apart from BAZ2A with a Naïve Bayes score of 0.44). Overall, the HCIPs identified with our custom database included the most confident HCIPs from the analysis done with the SwissProt database, but also highlighted novel interactions, creating a filtered yet interconnected network (Fig. 4D). Comparing the networks generated with the SwissProt or our custom database, this latest network displayed very similar characteristics with a degree distribution consistent with that of known protein–protein networks and retained interconnectivity (Fig. 4D). The network displayed a similar distribution of shortest path lengths (average of 3.21 as for the network with the SwissProt database), and maintained an even distribution of clustering coefficients. This suggests that using targeted custom protein databases allows for specific enrichment of protein complexes.

### 3.5 Deeper ORF annotation highlights novel interactors of viral proteins

**Fig. 5:**
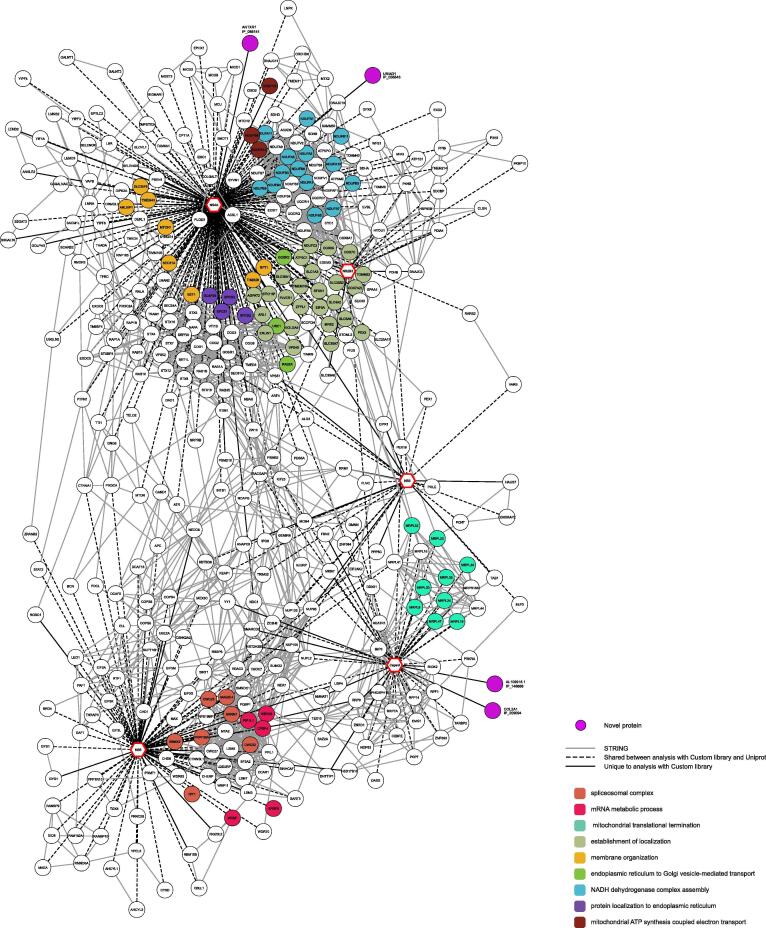
Network of viral-host protein–protein interactions built from the custom database Network of protein interactions for proteins of the Zika virus (indicated as octogonal nodes with thick red borders) with proteins with a degree higher than 1. The nodes correspond to canonical human proteins (circle with black borders) or alternative proteins (purple circle) identified by AP-MS data analysis. Nodes of canonical proteins are coloured based on their associated gene ontology as indicated on the bottom right corner. Edges represent confident interactions between two proteins as follows: solid black lines are interactions only identified in the current analysis; dashed black lines are interactions shared with the original study (Shah, *et al*.); and full grey lines are host–host protein interactions retrieved from the STRING database. (For interpretation of the references to color in this figure legend, the reader is referred to the web version of this article.)

We reconstructed the viral-host protein interaction network from the analysis using our custom database (Fig. 5, Suppl. Fig. 4). For the capsid, NS5 and NS4A viral proteins, our tailored analysis identified proteins for the vast majority also identified with the SwissProt database (67.2%, 81.5% and 82.4% respectively). Interestingly, we identified four novel proteins (purple nodes, Fig. 5) confidently interacting with viral proteins. Two alternative proteins (IP_209094 and IP_148668, accessions from the OpenProt resource) were found interacting with capsid, and two others (IP_086141 and IP_058843) were found interacting with NS4A.

**Fig. 6:**
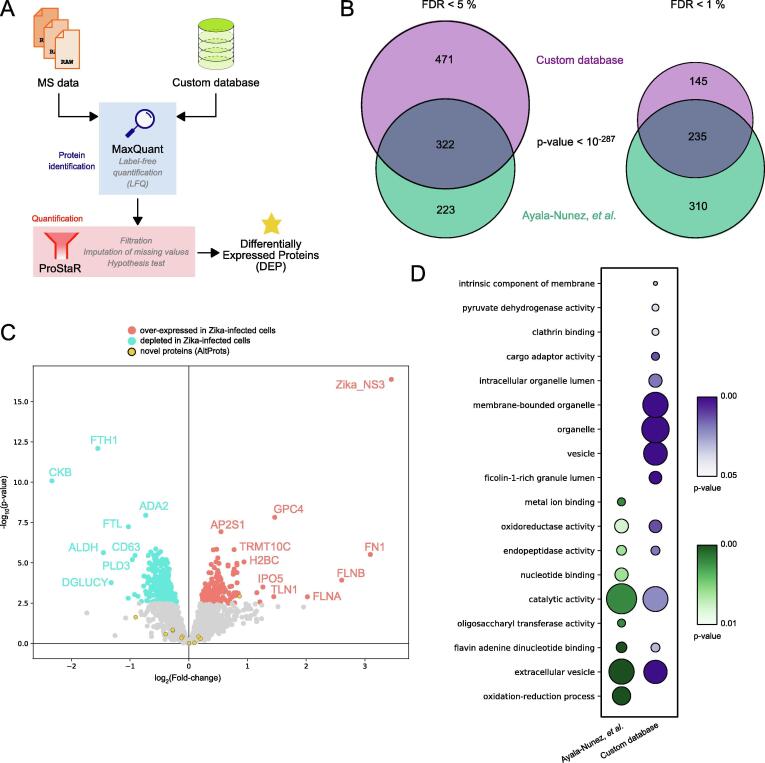
Proteome profiling with the custom database identifies novel proteins A. Graphical representation of the pipeline used for quantitative proteome profiling analysis. Mass spectrometry (MS) data is interrogated using the custom database defined in Fig. 3. The pipeline contains a step of protein identification (blue) using MaxQuant, followed by protein quantification (red) using ProStaR, to produce a list of differentially expressed proteins (DEPs). B. Overlap of proteins identified in this analysis (custom database) with that of the original study from Ayala-Nunez and colleagues [9]. Significance of the overlap was evaluated using a Fisher’s exact test. C. Volcano plot of differentially expressed proteins during Zika virus infection. Proteins found significantly upregulated in the Zika-infected conditions are indicated in red, while those found significantly down-regulated are indicated in blue. Novel proteins are highlighted in yellow with a black edge. D. Gene ontology (GO) enrichment of significantly differentially expressed proteins. All significant enrichments (FDR < 1%, Benjamini-Hochberg correction) are listed across both analyses. The dot size relates to the number of proteins supporting this GO term enrichment, while the color relates to the p-value. (For interpretation of the references to color in this figure legend, the reader is referred to the web version of this article.)

The alternative protein IP_209094 originates from messenger RNAs (mRNA) from the *COL2A1* gene. According to the OpenProt resource, this protein is 54 amino acids long and has been detected in two proteomics studies on NCI-60 cancer cell line panel. One study looked at the global proteome (PRDB000035), while the other looked at extracellular vesicles (PXD005479). The protein is conserved across 3 species (IP_209094). We confidently identified this alternative protein with the unique peptide MVKLENLEKLVK (Suppl. Fig. 5A), not shared with any other known protein. Although this protein does not contain any predicted functional domain on OpenProt, the DeepGOPlus tool found similarities with proteins involved in viral processes and ribosome biogenesis [61]. This is in line with other proteins interacting with the capsid in our analysis and others who reported known ribosomal proteins interacting with the capsid [7], [8], [11].

The IP_148668 alternative protein also interacts with the capsid. The protein originates from a ncRNA associated with *AL109918.1* (or *LOC730101*), a gene annotated as non-coding and associated with cancer [62]. This alternative protein displays both MS-based and ribosome profiling-based evidence on the OpenProt resource (MS score of 2 and translation score of 3). We confidently identified this alternative protein with the unique peptide CLTLPFVSPMNQSWDTSKK, not shared with any other known protein. Although OpenProt does not list any identified predicted functional domain, the latest CDD annotation identifies a RNA recognition motif (accession cd12757) [63].

The alternative protein IP_058843, interacts with the NS4A viral protein. This alternative protein was confidently identified with a unique peptide, QSVVLLSSSRR (Suppl. Fig. 5B). Interestingly, this protein is encoded in the 3′ untranslated region (UTR) of the *UBIAD1* gene. The protein is 80 amino acids long, is conserved in the chimpanzee (*Pan troglodytes*), and has already been detected once according to the OpenProt resource.

The alternative protein IP_086141 interacts with the NS4A viral protein. This alternative protein was confidently identified with a unique peptide (Suppl. Fig. 5C). Interestingly, this protein originates from the *ANTXR1* gene and its ORF overlaps the ANTXR1 canonical ORF in a different reading frame (Suppl. Fig. 5D). The protein is 78 amino acids long and is conserved between chimpanzee (*Pan troglodytes*), mouse (*Mus musculus*), cow (*Bos Taurus*), and sheep (*Ovis aries*) (Suppl. Fig. 5E). It has been detected once by MS according to the OpenProt resource (PXD011929) and was also detected in a study in HeLa cells nuclei [64].

As demonstrated here, deeper ORF annotation and the use of transcriptomic-informed database allow for the identification of novel proteins interacting with Zika viral proteins.

### 3.6 Deeper ORF annotations highlight novel differentially expressed proteins

Additionally, as it has been demonstrated before with other viruses, the translational changes upon Zika virus infection could lead to differentially expressed novel proteins [17], [18], [20]. We retrieved a publicly available dataset of quantitative proteome profiling upon Zika virus infection in monocytes [9]. We used the same pipeline as in the original study but queried the MS data with our custom database (Fig. 6A). When compensating for multiple comparisons, the original study adjusted the p-value for a FDR between 1% and 5%, while we fixed the FDR at 5% or 1%. Using the FDR set to 5%, we identified 793 differentially expressed proteins between the control and the Zika-infected samples (Fig. 6B). Out of these, 322 proteins were also identified as differentially expressed in the original study (Suppl. Table 6). This corresponds to a highly significant overlap of 59.1% (Fisher’s exact test, p-value < 10^-287^). With the FDR set to 1%, we identified 380 differentially expressed proteins (DEPs), from which 235 were also identified in the original study. This 1% FDR filtering thus corresponded to a highly significant overlap of 43.2% with the original dataset (Fisher’s exact test, p-value < 10^-287^, Suppl. Table 6). For downstream analyses, we used this more confident set of DEPs filtered at a 1% FDR. We identified a total of 138 up-regulated proteins and 242 down-regulated proteins (Fig. 6C).

We confidently identified 12 alternative proteins (IP_068551, IP_070279, IP_074954, IP_139806, IP_157897, IP_195829, IP_216771, IP_232994, IP_233268, IP_240469, IP_265139, and IP_276654 - https://openprot.org/p/savedSearch/lCa). One of these proteins, IP_265139, encoded in an ORF overlapping that of the ATP5F1A protein, was found significantly up-regulated in Zika-infected samples (Fig. 6C).

Furthermore, our analysis with a custom database identified a majority of GO terms enriched in the original analysis (5 out of 9, Fig. 6D). Although, we did not identify GO terms relating to oxidation–reduction or nucleotide binding enriched, we did identify terms relating to vesicle formation and granule lumen that were not reported in the original study (Suppl. Table 7). This suggests that the use of a custom database might identify enrichment of more specific terms.

## 4 Discussion

In this study, we explored publicly available datasets of pathogen-host interactions to further analytical methods aimed at the production of more powerful models of these biological systems. We demonstrated the importance of protein groups handling when comparing MS datasets and protein interaction networks. Furthermore, using a custom database we were able to retrieve biologically relevant protein complexes, and discover alternative proteins as novel interactors of Zika viral proteins. Although more bench work is needed to functionally characterize the identified alternative proteins and validate the interactions with viral proteins, this study lends support to the use of transcriptomic-informed databases in proteomics. The computational framework presented here highlights the biological insights gained from using deeper ORF annotations [17], [19], [20], [21], [65], [66]. The application of this framework on published proteomics dataset might shed light on cellular processes previously not considered [26], [30], [32], [67]. Such proteogenomics-like endeavour is facilitated by existing repositories of experimentally supported yet non-annotated ORFs, such as the OpenProt resource or the sORFs repository [28], [30].

The discovery of unannotated ORFs as differentially expressed proteins (DEPs) or highly confident interacting proteins (HCIPs) upon viral infection has previously been shown with the human cytomegalovirus, retroviruses and flu lysates [17], [19], [20]. Some of the identified alternative proteins originated from pseudogenes, which should be re-classified as paralogs. Various pseudogenes were previously shown to produce functional proteins, and many have been involved in human diseases [68], [69], [70], [71]. Moreover, the evolutionary arms race in which virus and host are involved imposes pressure for constant evolution of immune evasion or interaction strategies. Thus, the evolution strategies of viral-host protein interactions involve gene duplication (paralogy) [72], [73], [74]. Although mostly studied in the viral genome, this paralogy strategy is not exclusive to the virus [74]. This suggests that many alternative proteins from pseudogenes might be important in viral-host protein interaction networks.

Furthermore, using publicly available RNA-seq datasets, a custom protein database can be created which enriches biologically relevant identifications. Both the sORFs repository and the OpenProt resource allow custom downloads of novel ORFs [30], [75]. The use of customized databases optimized for the size of the search space and the biological relevance of included proteins have been shown to outperform standard database strategies [76], [77]. The results presented in this study show that biologically relevant information can be gained from the use of a custom protein database. Although the most confident protein identifications were reported by analyses with both the SwissProt database and the custom database, the latter provided additional identifications leading to an enrichment in GO terms relevant for viral processes and supported by previous experimental evidence [14], [78], [79].

Notably, we retrieved an enrichment in proteins known to associate with the nuclear chromatin in the capsid interactors (Fig. 4). These are proteins involved in the neuronal development [80], [81], [82], [83], [84]. Interestingly, the capsid is the only Zika viral protein reported to associate with proteins involved in the neuronal development [3]. These proteins represent important interactors to better understand ZIKV-mediated microcephaly. Interestingly, in the original study and other datasets, nucleolin (NCL) was identified as strong capsid interactor [3], [7], [8]. However, in our analysis, NCL was indeed identified but did not pass filters due to a lack of specificity and low abundance (Suppl. Table 5).

Our strategy identified biologically relevant canonical proteins and novel proteins. However, the RNomics and proteomics datasets in this study were not paired and displayed differences in cellular background and infection stage. This might prevent the identification of other biologically relevant proteins; on the other hand, it also indicates that the identified interactors show a high degree of conservation across cellular backgrounds. Using paired data might lead to other protein identifications, although maybe more specific to a cellular background, experimental design or infection stage [3]. Furthermore, the AP-MS dataset used here over-expressed viral proteins individually which may prevent the detection of biologically relevant protein that necessitate the combined effect of all viral proteins.

## CRediT authorship contribution statement

**Sebastien Leblanc:** Software, Formal analysis, Visualization. **Marie A. Brunet:** Conceptualization, Methodology, Software, Validation, Formal analysis, Visualization, Resources, Writing - original draft.

## Declaration of Competing Interest

The authors declare that they have no known competing financial interests or personal relationships that could have appeared to influence the work reported in this paper.

## Appendix A Supplementary data

Supplementary data 1

Supplementary data 2 Comparison of our analysis with the original study. A. Overlap of all proteins identified (prior to HCIP filtering) with the SwissProt database (blue) or in the original study (green) by Shah and colleagues. B. Overlap of all interactions identified (prior to HCIP filtering) with the SwissProt database (blue) or in the original study (green) by Shah and colleagues. C. Overlap of highly confident protein interactions (HCIPs) identified with the SwissProt database (blue) or in the original study (green) by Shah and colleagues, following filtering with MiST alone (threshold at 0.75). D. Overlap of highly confident protein interactions (HCIPs) identified with the SwissProt database (blue) or in the original study (green) by Shah and colleagues, following filtering with CompPASS alone (threshold for the top 5 %). E. Curves representing on the left y-axis the precision (red), recall (green) and F1 score (black) according to the Naïve Bayes threshold. The blue curve represents the number of HCIPs called (mapped on the right y-axis). F. Table listing all model performance metrics for the 4 models considered: MiST alone, CompPASS alone, the union of MiST and CompPASS and the Naïve Bayes trained using the MiST and CompPASS scores as features (see material and methods). G. Distribution of the retrieved fraction of the 132 CORUM complexes shared between the SwissProt analysis (blue) and the original study (Shah in green).

Supplementary data 3 Comparison of the analysis with the custom database and with the SwissProt database and original study. A. Overlap of all proteins identified (prior to HCIP filtering) with the SwissProt database (blue) or the custom database (purple). B. Overlap of all interactions identified (prior to HCIP filtering) with the SwissProt database (blue) or the custom database (purple). C. Overlap of highly confident protein interactions (HCIPs) identified with the custom database (purple) or in the original study (green) by Shah and colleagues. D. Overlap of highly confident protein interactions (HCIPs) identified with the custom database (purple), the SwissProt database (blue) or in the original study (green) by Shah and colleagues. E. Curves representing on the left y-axis the precision (red), recall (green) and F1 score (black) according to the Naïve Bayes threshold. The blue curve represents the number of HCIPs called (mapped on the right y-axis).

Supplementary data 4 Complete viral-host protein interactions network using the SwissProt database. Complete network of protein interactions for proteins of the Zika virus (indicated as octogonal nodes with thick red borders). The nodes (circle with black borders) correspond to human proteins identified by AP-MS data analysis. Nodes are coloured based on their associated gene ontology as indicated on the bottom left corner. Edges represent confident interactions between two proteins as follows: solid black lines are interactions only identified in the current analysis; dashed black lines are interactions shared with the original study (Shah, et al.); and full grey lines are host-host protein interactions retrieved from the STRING database.

Supplementary data 5 Complete viral-host protein interactions network using the custom database. Network of protein interactions for proteins of the Zika virus (indicated as octogonal nodes with thick red borders). The nodes correspond to canonical human proteins (circle with black borders) or alternative proteins (purple circle) identified by AP-MS data analysis. Nodes of canonical proteins are coloured based on their associated gene ontology as indicated on the bottom right corner. Edges represent confident interactions between two proteins as follows: solid black lines are interactions only identified in the current analysis; dashed black lines are interactions shared with the original study (Shah, et al.); and full grey lines are host-host protein interactions retrieved from the STRING database.

Supplementary data 6 Novel proteins identified interacting with Zika viral proteins. A. Example of a MS/MS spectra for the alternative protein IP_058843 (protein accession from the OpenProt resource). Y-ions are annotated in red, b-ions in blue and others in green. The peptide sequence and its fragmentation is indicated at the top of the spectra. B. Example of a MS/MS spectra for the alternative protein IP_209094 (protein accession from the OpenProt resource). Y-ions are annotated in red, b-ions in blue and others in green. The peptide sequence and its fragmentation is indicated at the top of the spectra. C. Example of a MS/MS spectra for the alternative protein IP_086141 (protein accession from the OpenProt resource). Y-ions are annotated in red, and b-ions in blue. The peptide sequence and its fragmentation is indicated at the top of the spectra. Amino acids with post-translational modifications are coloured in yellow. D. Transcript and protein information at the ANTXR1 gene locus. The alternative protein IP_086141 (red), identified in panel E, is encoded in an ORF overlapping that of the canonical protein (green), but in a different reading frame. E. Conservation of IP_086141 across species (chimpanzee, mouse, cow and sheep). Residues are coloured based on the degree of identity (dark red for well conserved, to white for not conserved). Orthologous proteins were identified using the OpenProt resource. The peptide identified in panel E is indicated in bold.

Supplementary data 7 Control of data distribution after imputation of missing values. A. Distribution of protein mean intensities in the Mock condition before imputation of missing values (blue) and after imputation (red). B. Distribution of protein mean intensities in the Zika condition before imputation of missing values (blue) and after imputation (red).

Supplementary data 8 Output of the AP-MS pipeline of analysis with the SwissProt database, with MiST, CompPASS and Naive Bayes classifier outputs. List of proteins identified with the SwissProt database alongside their MiST, CompPASS and Naïve Bayes scores. The headers correspond to the Zika protein (Bait), the identified protein accession (Prey) and its gene name (Prey Gene), the MiST score (MiST), CompPASS Z-score (CompPASS Z) and Naïve Bayes score (Naïve Bayes). Alongside these metrics, the last column indicates whether that specific protein was identified in the original study.

Supplementary data 9 List of GO terms enrichment identified with the SwissProt database. List of GO terms identified with the SwissProt database. The headers correspond to the Zika protein (bait), GO term domain (Name Space), the GO term identification (GO term), the GO term description (Description), the p-value corrected with Benjamini-Hochberg (P-value) and the genes associated to this term (Genes).

Supplementary data 10 Transcripts expression level (Cufflinks output). This table corresponds to the Cufflinks output for the RNA-seq analysis.

Supplementary data 11 Output of the AP-MS pipeline of analysis with the custom database, with MiST, CompPASS and Naive Bayes classifier outputs. List of proteins identified with the custom database alongside their MiST, CompPASS and Naïve Bayes scores. The headers correspond to the Zika protein (Bait), the identified protein accession (Prey) and its gene name (Prey Gene), the MiST score (MiST), CompPASS Z-score (CompPASS Z) and Naïve Bayes score (Naïve Bayes). Alongside these metrics, the last two columns indicates whether that specific protein was identified in the SwissProt analysis (shared with Control) or in the original study (shared with Shah).

Supplementary data 12 List of GO terms enrichment identified with the custom database. List of GO terms identified with the custom database. The headers correspond to the Zika protein (bait), GO term domain (Name Space), the GO term identification (GO term), the GO term description (Description), the p-value corrected with Benjamini-Hochberg (P-value) and the genes associated to this term (Genes).

Supplementary data 13 List of differentially expressed proteins, at a 1% FDR or a 5% FDR. Lists of the proteins identified in the whole proteome analysis filtered at a FDR of 1 % or 5 %. In each spreadsheet, the headers correspond to the protein accessions (Protein_IDs), the calculated log of the fold-change (logFC), the calculated p-value with a Benjamini-Hochberg correction for multiple comparison (P-Value). The last column is a boolean test to indicate whether the protein is significantly differentially expressed at the given FDR.

Supplementary data 14 List of GO terms enrichment identified within the differentially expressed proteins. List of GO terms identified in the original study of the whole proteome and in the re-analysis with our custom database. The headers correspond to the GO term identification, the p-value and the number of genes supporting this GO term in the original publication, the p-value and the number of genes supporting this GO term in our re-analysis with our custom database, and the description of the GO term.
