# Supplementary figures and images for: Modelling of pathogen-host systems using deeper ORF annotations and transcriptomics to inform proteomics analyses
Source: Comput Struct Biotechnol J. 2020 Oct 14;18:2836–50. doi: 10.1016/j.csbj.2020.10.010 (PMC7585943; doi:10.1016/j.csbj.2020.10.010)

Supplementary Figure 1

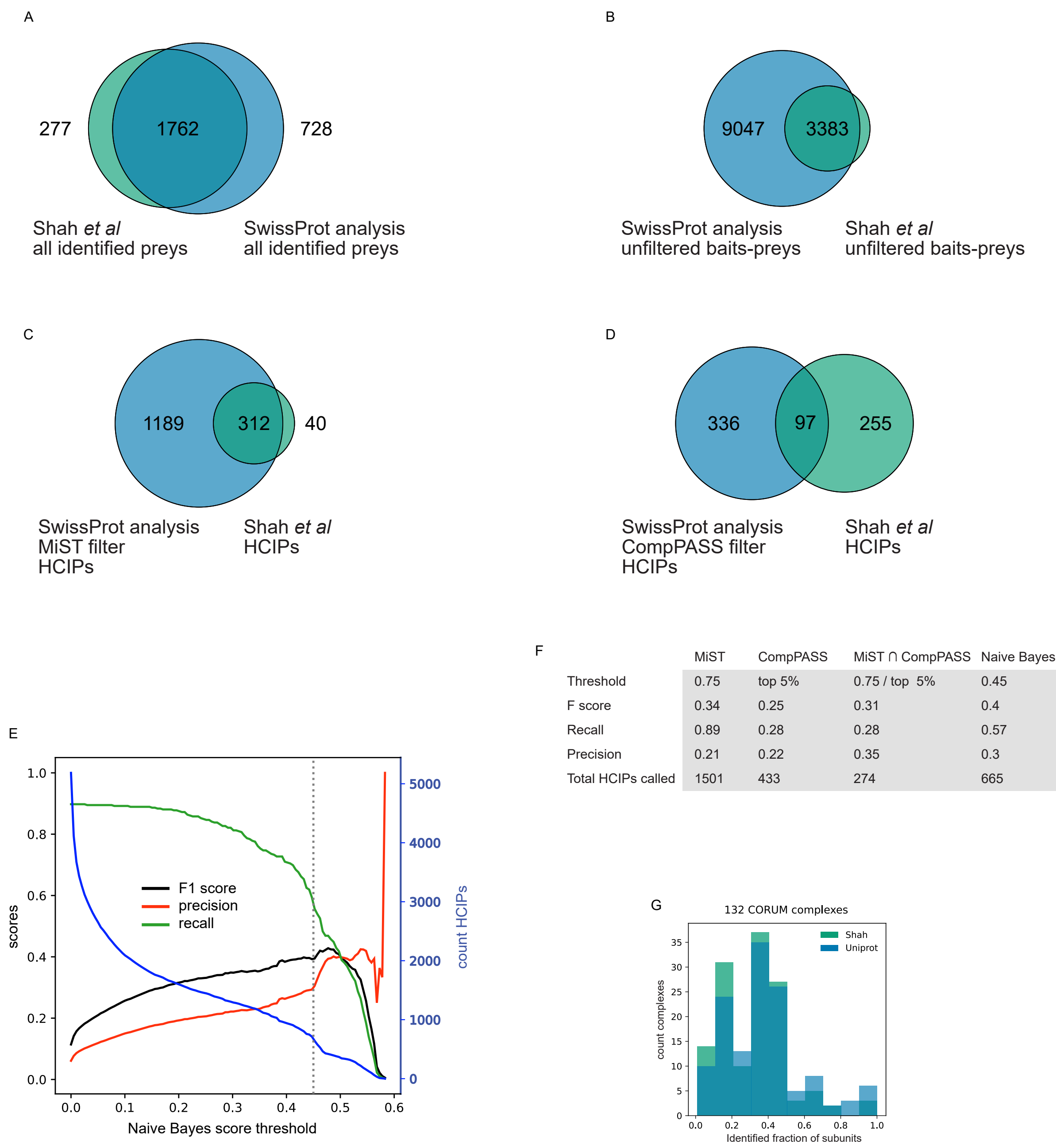

Supplement: Supplementary data 2 — Comparison of our analysis with the original study. A. Overlap of all proteins identified (prior to HCIP filtering) with the SwissProt database (blue) or in the original study (green) by Shah and colleagues. B. Overlap of all interactions identified (prior to HCIP filtering) with the SwissProt database (blue) or in the original study (green) by Shah and colleagues. C. Overlap of highly confident protein interactions (HCIPs) identified with the SwissProt database (blue) or in the original study (green) by Shah and colleagues, following filtering with MiST alone (threshold at 0.75). D. Overlap of highly confident protein interactions (HCIPs) identified with the SwissProt database (blue) or in the original study (green) by Shah and colleagues, following filtering with CompPASS alone (threshold for the top 5 %). E. Curves representing on the left y-axis the precision (red), recall (green) and F1 score (black) according to the Naïve Bayes threshold. The blue curve represents the number of HCIPs called (mapped on the right y-axis). F. Table listing all model performance metrics for the 4 models considered: MiST alone, CompPASS alone, the union of MiST and CompPASS and the Naïve Bayes trained using the MiST and CompPASS scores as features (see material and methods). G. Distribution of the retrieved fraction of the 132 CORUM complexes shared between the SwissProt analysis (blue) and the original study (Shah in green). [file mmc2.pdf]

Supplementary Figure 2

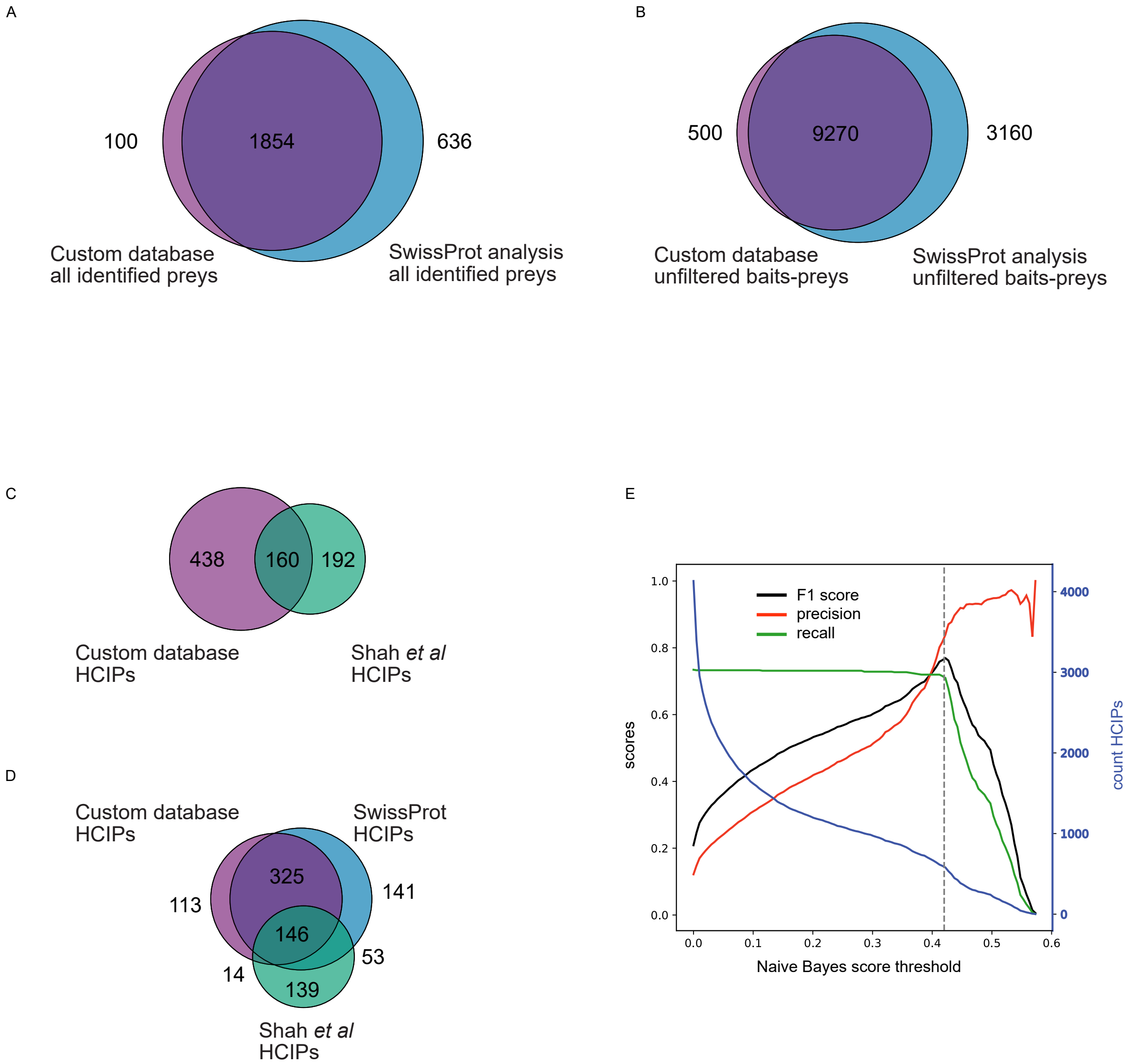

Supplement: Supplementary data 3 — Comparison of the analysis with the custom database and with the SwissProt database and original study. A. Overlap of all proteins identified (prior to HCIP filtering) with the SwissProt database (blue) or the custom database (purple). B. Overlap of all interactions identified (prior to HCIP filtering) with the SwissProt database (blue) or the custom database (purple). C. Overlap of highly confident protein interactions (HCIPs) identified with the custom database (purple) or in the original study (green) by Shah and colleagues. D. Overlap of highly confident protein interactions (HCIPs) identified with the custom database (purple), the SwissProt database (blue) or in the original study (green) by Shah and colleagues. E. Curves representing on the left y-axis the precision (red), recall (green) and F1 score (black) according to the Naïve Bayes threshold. The blue curve represents the number of HCIPs called (mapped on the right y-axis). [file mmc3.pdf]

Supplementary Figure 3

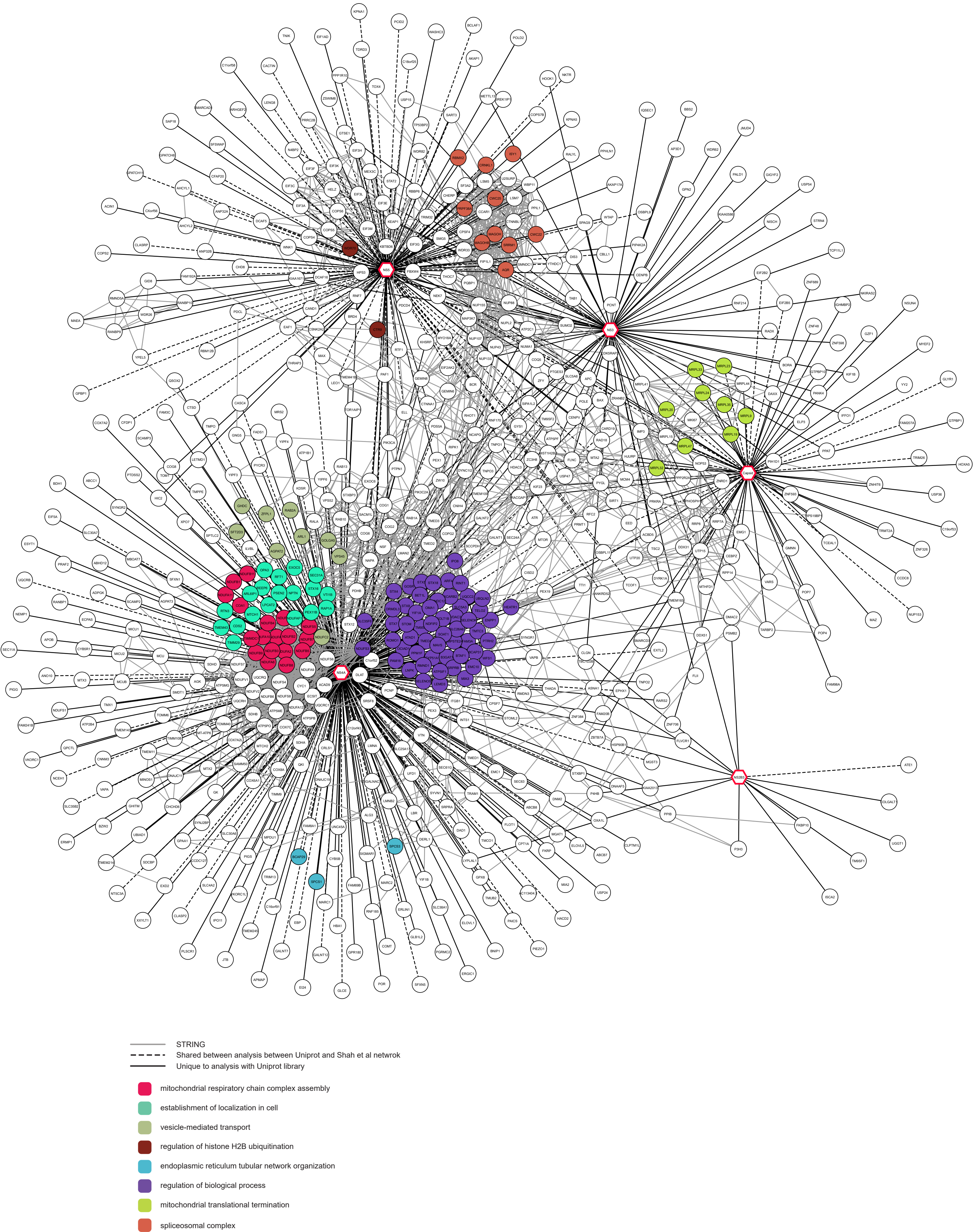

Supplement: Supplementary data 4 — Complete viral-host protein interactions network using the SwissProt database. Complete network of protein interactions for proteins of the Zika virus (indicated as octogonal nodes with thick red borders). The nodes (circle with black borders) correspond to human proteins identified by AP-MS data analysis. Nodes are coloured based on their associated gene ontology as indicated on the bottom left corner. Edges represent confident interactions between two proteins as follows: solid black lines are interactions only identified in the current analysis; dashed black lines are interactions shared with the original study (Shah, et al.); and full grey lines are host-host protein interactions retrieved from the STRING database. [file mmc4.pdf]

Supplementary Fiure 4

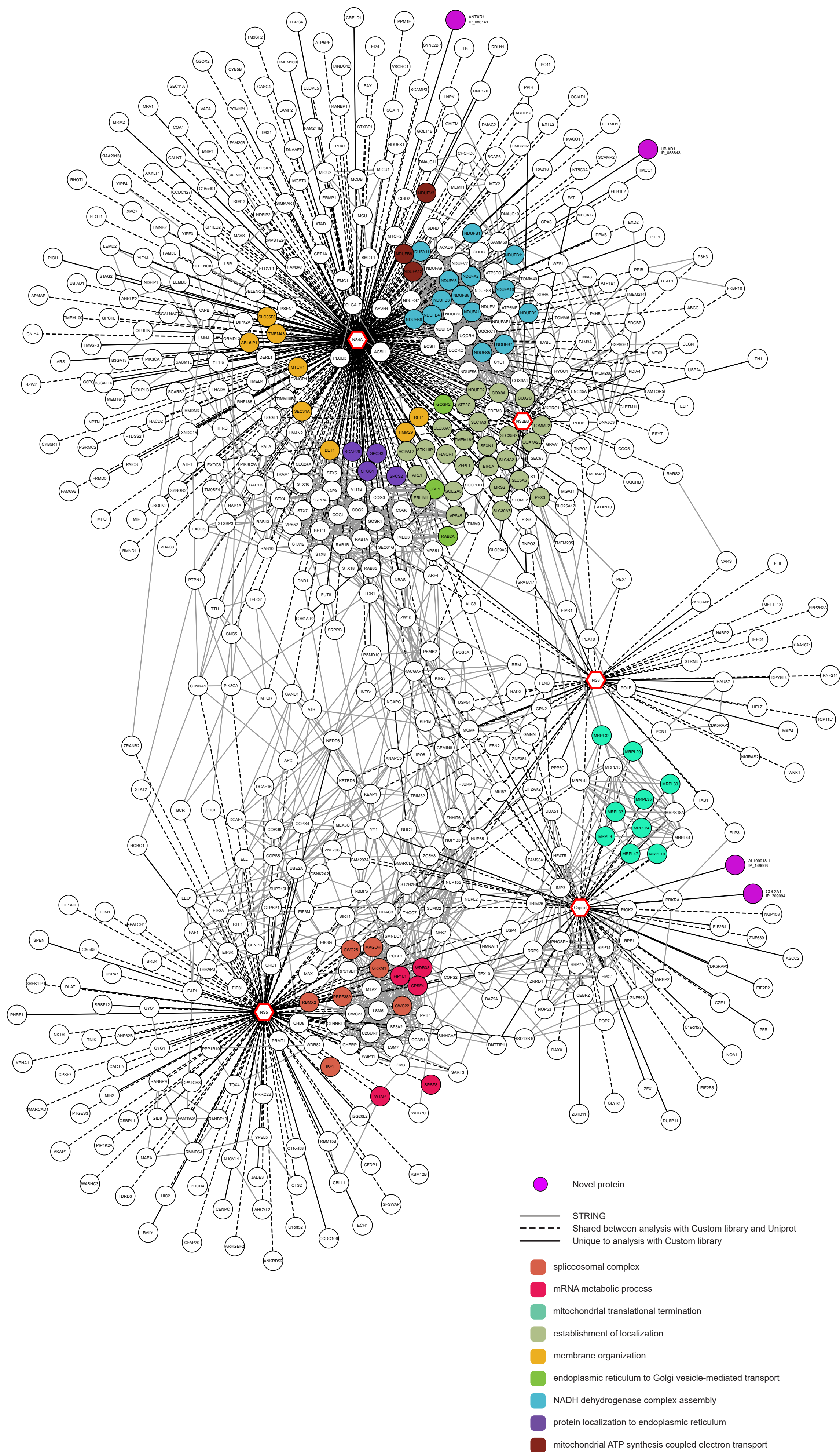

Supplement: Supplementary data 5 — Complete viral-host protein interactions network using the custom database. Network of protein interactions for proteins of the Zika virus (indicated as octogonal nodes with thick red borders). The nodes correspond to canonical human proteins (circle with black borders) or alternative proteins (purple circle) identified by AP-MS data analysis. Nodes of canonical proteins are coloured based on their associated gene ontology as indicated on the bottom right corner. Edges represent confident interactions between two proteins as follows: solid black lines are interactions only identified in the current analysis; dashed black lines are interactions shared with the original study (Shah, et al.); and full grey lines are host-host protein interactions retrieved from the STRING database. [file mmc5.pdf]

Figure 6

A

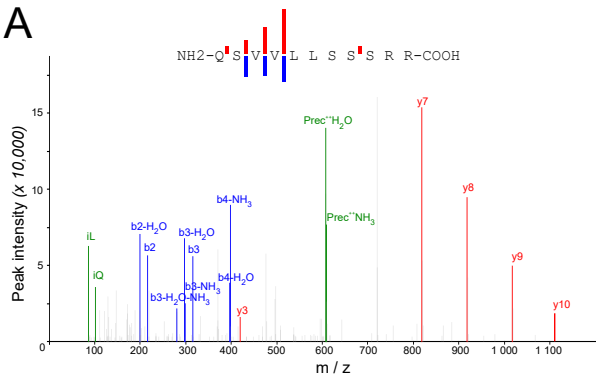

B

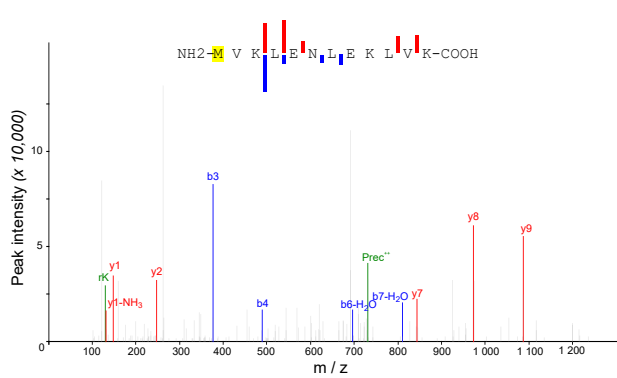

C

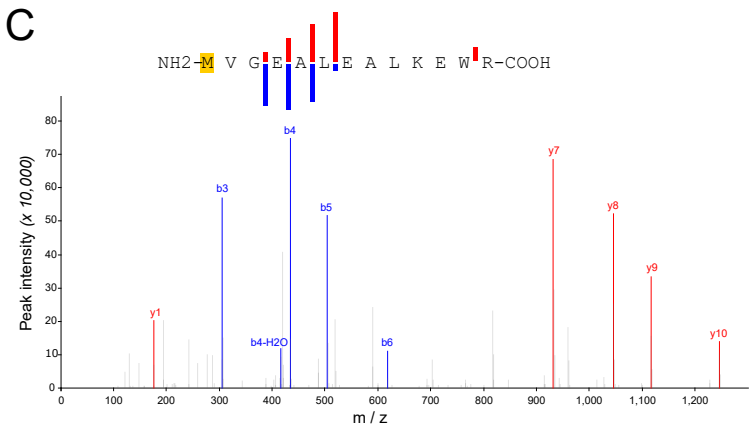

D

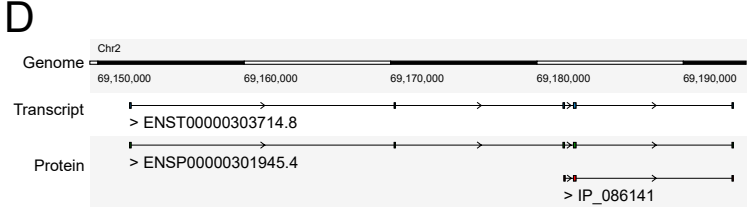

E

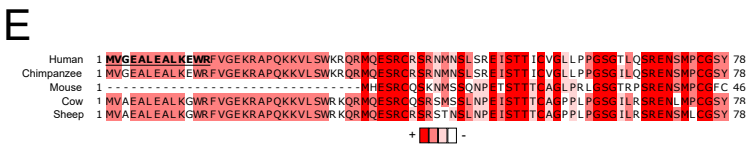

Supplement: Supplementary data 6 — Novel proteins identified interacting with Zika viral proteins. A. Example of a MS/MS spectra for the alternative protein IP_058843 (protein accession from the OpenProt resource). Y-ions are annotated in red, b-ions in blue and others in green. The peptide sequence and its fragmentation is indicated at the top of the spectra. B. Example of a MS/MS spectra for the alternative protein IP_209094 (protein accession from the OpenProt resource). Y-ions are annotated in red, b-ions in blue and others in green. The peptide sequence and its fragmentation is indicated at the top of the spectra. C. Example of a MS/MS spectra for the alternative protein IP_086141 (protein accession from the OpenProt resource). Y-ions are annotated in red, and b-ions in blue. The peptide sequence and its fragmentation is indicated at the top of the spectra. Amino acids with post-translational modifications are coloured in yellow. D. Transcript and protein information at the ANTXR1 gene locus. The alternative protein IP_086141 (red), identified in panel E, is encoded in an ORF overlapping that of the canonical protein (green), but in a different reading frame. E. Conservation of IP_086141 across species (chimpanzee, mouse, cow and sheep). Residues are coloured based on the degree of identity (dark red for well conserved, to white for not conserved). Orthologous proteins were identified using the OpenProt resource. The peptide identified in panel E is indicated in bold. [file mmc6.pdf]

**A**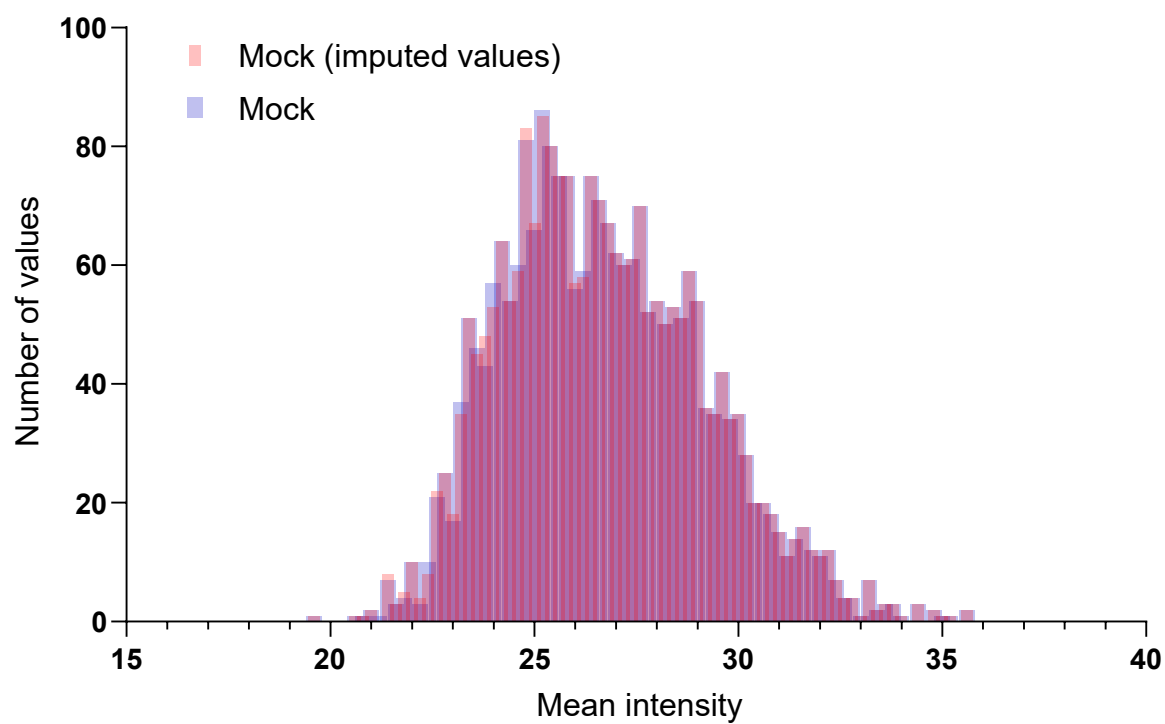**B**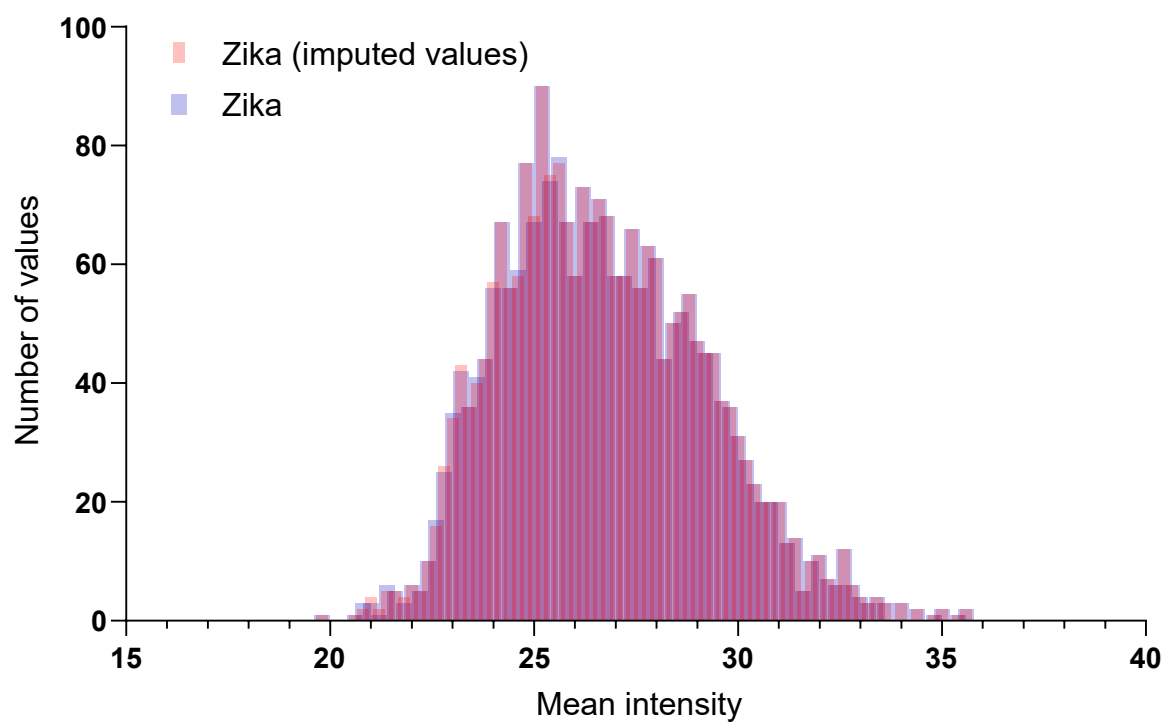

Supplement: Supplementary data 7 — Control of data distribution after imputation of missing values. A. Distribution of protein mean intensities in the Mock condition before imputation of missing values (blue) and after imputation (red). B. Distribution of protein mean intensities in the Zika condition before imputation of missing values (blue) and after imputation (red). [file mmc7.pdf]
